# Effect of the Side-Chain Length in Polycarboxylic Superplasticizer on the Competition Adsorption in the Presence of Montmorillonite: A Density Functional Theory Study

**DOI:** 10.3390/molecules29040752

**Published:** 2024-02-06

**Authors:** Zhihao He, Teng Huang, Meiben Gao, Desong Kong, Meng Li

**Affiliations:** 1 School of Emergency Management, Xihua University, Chengdu 610039, China; 2 State Key Laboratory of Geohazard Prevention and Geoenvironment Protection, Chengdu University of Technology, Chengdu 610059, China; 3 School of Environment and Resource, Southwest University of Science and Technology, Mianyang 621010, China; 4 Key Laboratory of Waste Treatment and Resource Recycle, Southwest University of Science and Technology, Ministry of Education, Mianyang 621010, China

**Keywords:** PCEs, compatibility, clay, cement concrete, molecular simulation

## Abstract

Polycarboxylic superplasticizers (PCEs) exhibit numerous advantages as concrete additives, effectively improving the stability and strength of concrete. However, competitive adsorption of PCEs occurs in the presence of clay, which may affect the cement dispersion and water-reducing performance. Extensive research has been conducted on the physical and mechanical properties of PCEs; however, the effect of the diverse structures of PCEs on the competitive adsorption on clay and cement hydration products has been rarely studied. This study employs Ca-montmorillonite (CaMMT) as a clay representative, by constructing adsorption models of PCEs on CaMMT and cement hydration products. A comparison of the adsorption energies considering different side-chain lengths of PCEs is included. Typically, the adsorption energy on CaMMT is lower than that on hydration products, leading PCEs to preferentially adsorb on the clay, thereby reducing its effective dosage in the cement particles. The challenge of PCE adsorption on CaMMT increases with the polymerization degree, and methylallyl polyoxyethylene ether (HPEG) exhibits lower adsorption energies on CaMMT. The density of states (DOS) analysis indicated the highest peak values of allyl polyethylene ether (APEG) as well as the peak area at n (polymerization degree) = 1. The total number of transferred electrons for APEG was 0.648, surpassing those of other PCEs. The interaction mechanism of PCEs with clay and hydration products is further elucidated through electronic gain/loss analysis, also providing a basis for the theoretical analysis on how to reduce the adsorption of PCEs on clay and the structural design of mud-resistant PCEs.

## 1. Introduction

**Figure 1:**
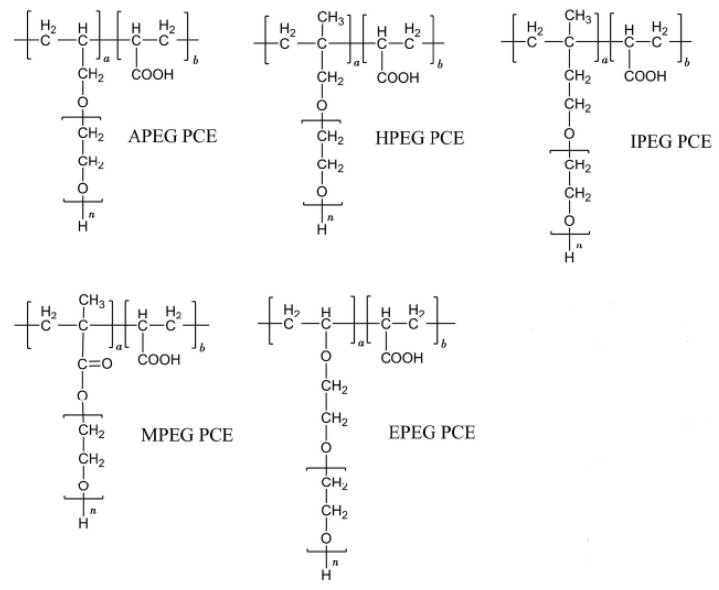
Structures of different types of PCEs.

Polycarboxylic superplasticizers (PCEs) have a typical comb-like structure formed of the side chain and main chain of polyoxyethylene (polyethylene glycol). Owing to the structure and composition of the large monomer, different types of PCEs can be obtained by copolymerization reactions with acrylic acid (Figure 1) [1,2].

For strong water reduction capabilities and excellent dispersion performance, PCEs find extensive application in large-scale engineering projects, such as hydroelectric dams, tunnels and bridges [3,4]. While PCEs are very sensitive to mud, primarily composed of montmorillonite, kaolin and other clay minerals are found in sand and gravel aggregates. The potent interaction between clay and PCEs can diminish the amount of water-reducing agent adsorbed by the cement, thereby reducing the dispersion efficiency and water-reducing performance of PCEs [5,6,7]. Tang Shengxuan (2018) [8] reported that clay strongly affects the water-reducing and dispersing effect of PCEs, so fresh cement can lose the working characteristics when the clay content is ≥5%. Moreover, diverse clay sources and their compositions could also change the cement dispersion performance in the presence of superplasticizers. Notably, montmorillonite, owing to the presence of more positive charges in the interlayer end face, exhibits a higher adsorption capacity for water-reducing agents compared to illite, kaolinite and cement [9,10,11]. Our previous studies also found that there is competitive adsorption between clay and hydration products when the admixture participates in the hydration reaction, establishing the following adsorption sequence for different minerals: montmorillonite > illite > calcite > orthoclase > quartz [12].

The effectiveness of various PCEs in dispersion hinges largely on their adsorption capacity on cement particles [13,14]. Consequently, variations in side-chain densities and main-chain lengths can impact the competitive adsorption of PCEs on both clay and cement particles. This, in turn, influences the crucial properties of cement, including fluidity and setting time [15,16,17]. Ouellet-Plamondon et al. (2020) [18] synthesized and characterized PCEs with diverse structures, revealing that distinctions in main- and side-chain characteristics alter the adsorption capacity, thereby affecting the dispersing and retarding effects. Erzengin S G et al. (2018) [19] observed that PCEs with lower side-chain densities enhance the strength and flowability of cement slurries in the presence of clay, whereas side chains with higher molecular weights improve dispersibility. However, PCE macromolecules readily intercalate and adsorb onto montmorillonite, potentially impacting their dispersion performance [19,20,21]. Tan Hongbo et al. (2017) [17] investigated the effect of sodium gluconate on the clay tolerance of PCE. The results show that sodium gluconate cannot be intercalated into montmorillonite, thereby altering the amount of PCE in the solution, which can affect the intercalation of S-PCE rather than directly hinder the intercalation. Existing research primarily centers on the physical and mechanical properties, such as the fluidity and setting time of cement, and scant attention has been given to the diverse structures of PCEs’ influencing competitive adsorption on clay and cement hydration products. By combining the experiments and molecular simulations, we could explore the difficulty order and influencing factors of competitive adsorption among various PCEs on clay and cement, further establishing the influence law of the compatibility considering the molecular structural parameters of PCEs [22,23].

The hydration reaction of cement results in the formation of Ca(OH)_2_ (CH), C-S-H gel and other products. As the typical crystalline phase hydration product in the cement hydration, CH particles’ micro-morphology also has a certain impact on the mechanical properties of cement [24,25]. Song Yufeng et al. (2023) [26] explored the mechanical properties and corrosion behaviors of CSA mortar with different ratios of Ca(OH)_2_. The optimal Ca(OH)_2_ ratio in the CSA/Ca(OH)_2_ composite is 6% in terms of its mechanical performance, with an increased quantity of Ca(OH)_2_, and the amount of some hydration phases, such as Al(OH)_3_, reduced, resulting from their reaction with Ca(OH)_2_. Building upon our prior investigations into the effects of distinct active groups and structures on the adsorption properties of CH [24], we investigate the competitive adsorption of various PCEs on the clay and cement, also with the interaction mechanism using molecular dynamic simulations. Research on the anti-clay modification and the structural design of PCEs will be further expanded by exploring factors that influence the interaction between the PCEs and clay [27,28].

## 2. Results and Discussion

### 2.1. Adsorption Energies of PCEs on CaMMT and CH

#### 2.1.1. APEGs with Different Side-Chain Polymerization Degrees

**Figure 2:**
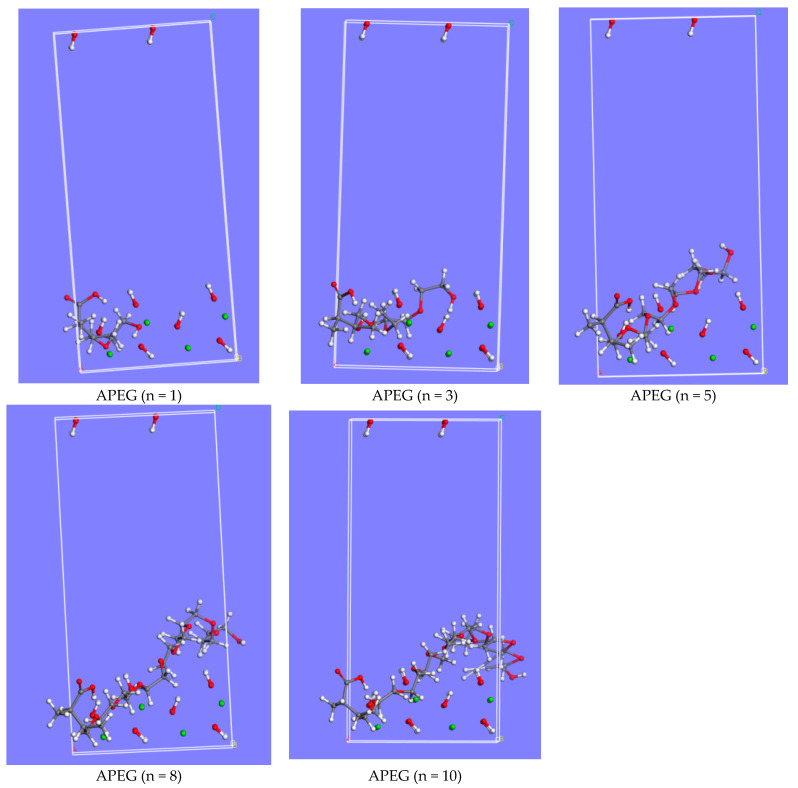
Adsorption models of CH (101) surface on different APEGs.

**Figure 3:**
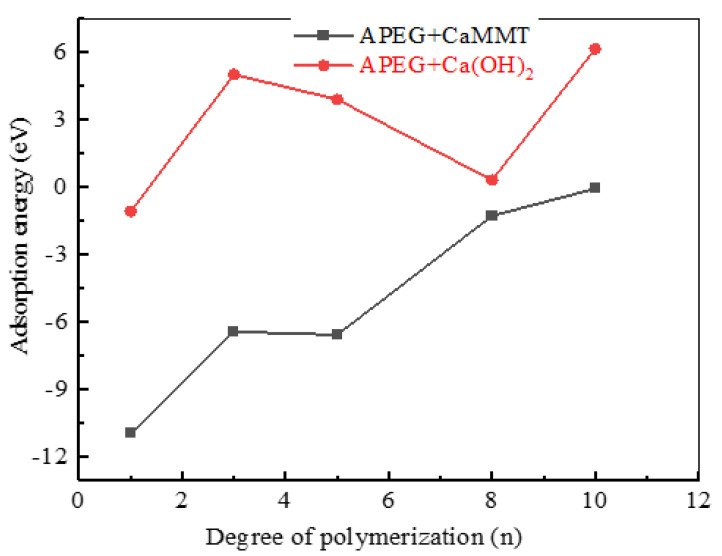
Comparison of the adsorption energies of APEGs with different side-chain polymerization degrees.

Using the hydration product CH as an illustration, Figure 2 presents adsorption models of APEGs with varying side-chain polymerization degrees on the CH (101) surface. A comparison of adsorption energies on CaMMT and CH is depicted in Figure 3.

The adsorption energy is −10.94 eV at n = 1, and it gradually rises on CaMMT as the polymerization degree increases, so adsorption is more difficult. The adsorption energy on CH is typically positive and considerably higher than that on CaMMT with the same degree of polymerization. This suggests a preference for APEGs to be adsorbed and consumed by CaMMT.

#### 2.1.2. IPEGs with Different Side-Chain Polymerization Degrees

**Figure 4:**
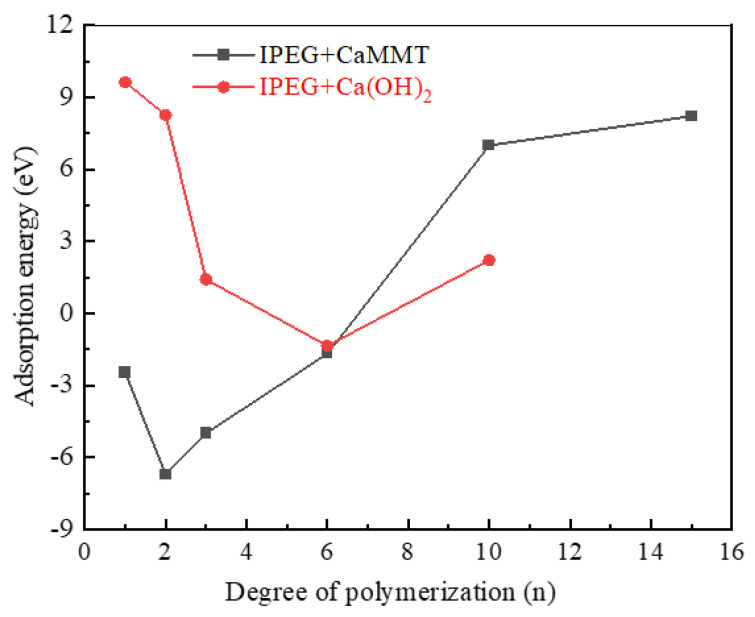
Comparison of the adsorption energies of IPEGs with different side-chain polymerization degrees.

A comparison of the adsorption energies of IPEGs with varying degrees of polymerization on CaMMT and CH is depicted in Figure 4.

The adsorption energy of IPEGs on CaMMT first decreases and then increases at low polymerization degrees, reaching its lowest value of −6.71 eV at n = 2. The adsorption energy then gradually rises as the polymerization degree increases (n ≥ 3), reaching a positive value at n = 10, so the adsorption on CaMMT may fail to proceed.

Additionally, the adsorption energy on CH gradually decreases as the polymerization degree increases, particularly at relatively low polymerization degrees (n ≤ 6). The adsorption energy is 9.63 eV at n = 1, and it may decrease to a negative value (−1.35 eV) at n = 6; hence, the adsorption may be more likely to occur. Comparing the adsorption energies on CaMMT and CH with the same polymerization degree, when n ≤ 6, IPEG demonstrates a preference for adsorption on CaMMT. This preference is more pronounced at lower degrees of polymerization.

#### 2.1.3. HPEGs with Different Side-Chain Polymerization Degrees

**Figure 5:**
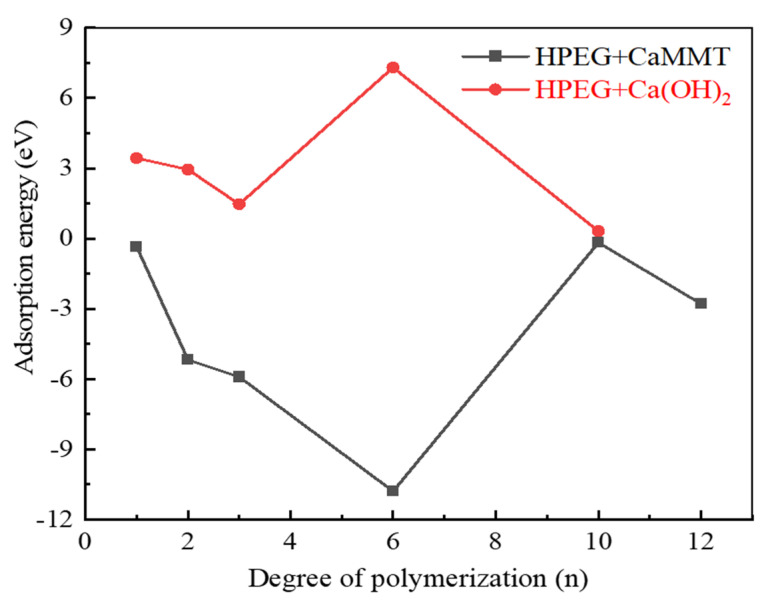
Comparison of the adsorption energies of HPEGs with different side-chain polymerization degrees.

Figure 5 presents a comparison of the adsorption energies of HPEGs with different polymerization degrees on CaMMT and CH.

At n = 6, the adsorption energy is −10.79 eV on CaMMT, significantly lower than that of other PCEs with the same degree of polymerization, signifying the easier adsorption of HPEGs. Even at n ≥ 10, the adsorption energy remains negative, indicating the possibility of adsorption on CaMMT, even at relatively high polymerization degrees. The adsorption energies of HPEGs on CH are always positive, as calculated in the text (n ≤ 10). With a lower adsorption energy, HPEG exhibited preferential adsorption on CaMMT rather than on the hydration products.

Therefore, by comparing the adsorption energies on CaMMT and CH, we can further discern the preferential adsorption behavior of PCEs. As the adsorption energy of HPEGs is usually lower than that of other types of PCEs, the adsorption on CaMMT is more prone to occur.

### 2.2. Electronic Structures and Charge Distribution Analysis

Utilizing DFT calculations enables the determination of microstructural details, including the density of states (DOS), molecular orbitals, charge distribution, etc. [29,30,31]. Building upon our prior findings [12,24], we employed the total density of states (TDOS) for analyzing electronic distribution and structures. Through a comparative study of changes before and after adsorption, we delved into the microscopic perspective of the interaction between PCEs and clay [32,33,34].

#### 2.2.1. DOS of Different PCEs before and after Adsorption

**Figure 6:**
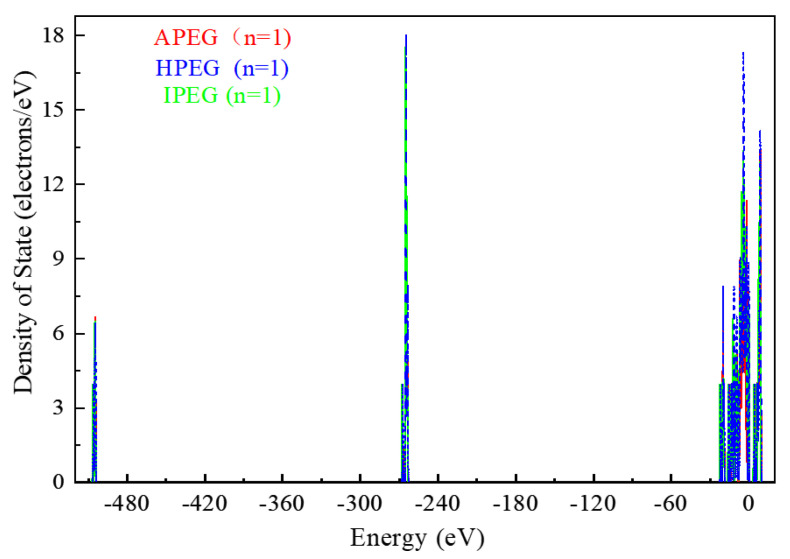
DOS analysis of different PCEs.

Assuming a polymerization degree of n = 1, the DOS analysis of different PCEs is shown in Figure 6. The ordinate axis value (DOS) of each PCE may concentrate in a range from −23 to 11 eV, which may display the majority of electron distribution. The peak area around −505 eV and −265 eV is extremely small, and the electron distribution is correspondingly lower.

To streamline this study, the subsequent discussion focuses exclusively on the adsorption models within a range from −23 to 11 eV.

**Figure 7:**
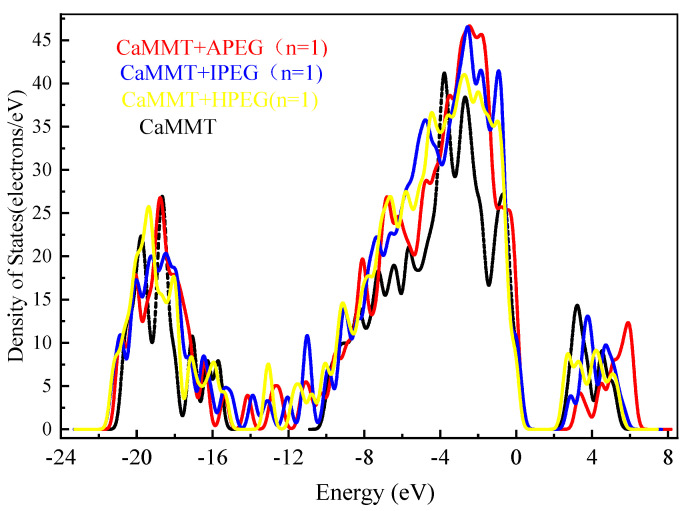
DOS analysis of CaMMT before and after the adsorption of different PCEs.

DOS analysis of CaMMT before and after the adsorption of different PCEs is shown in Figure 7. The value of the vertical axis gradually increased after adsorption. The initial value was 1.5 × 10^−4^ electrons/eV in APEG, which was higher than that before adsorption (1.0 × 10^−4^ electrons/eV). The peak values for APEG and IPEG were 46.65 and 46.55 electrons/eV, respectively, surpassing their values before adsorption. Additionally, the peak area was larger for APEG, indicating a more extensive electron distribution.

#### 2.2.2. Charge Distribution of Different Atoms in PCEs

**Figure 8:**
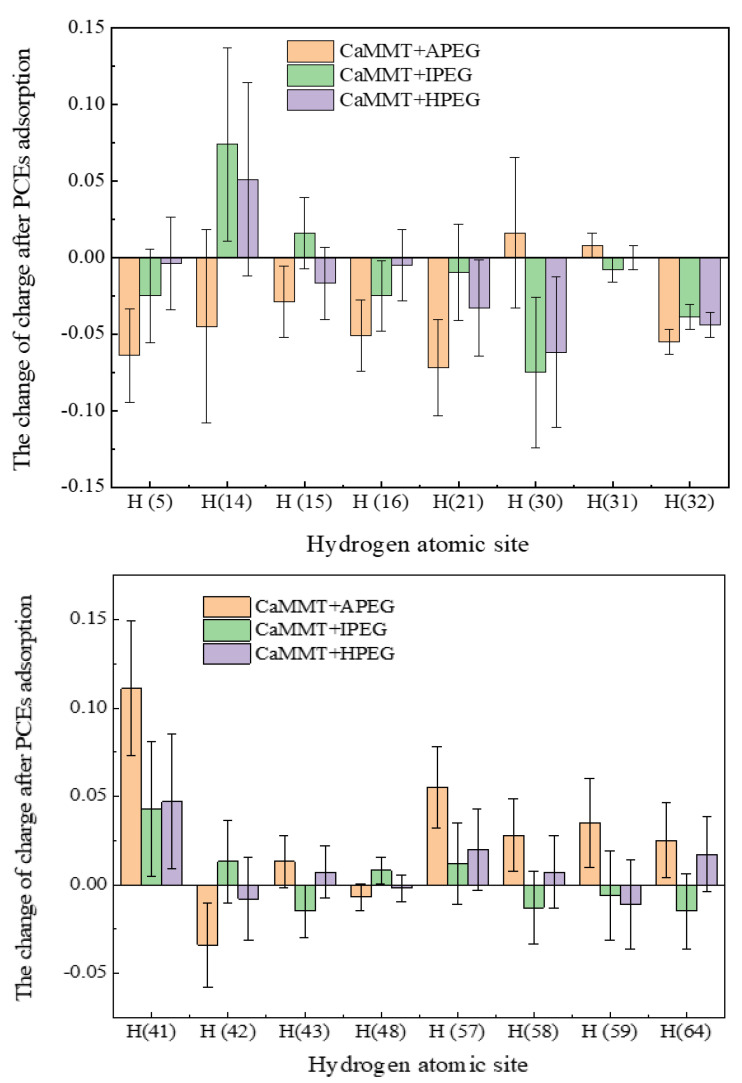
Statistical analysis of the charge of H atoms in CaMMT after PCEs adsorption.

In our earlier analysis of electronic distribution and structures, we illustrated the specific charge distribution of different atoms through Mulliken charge populations [12,35,36]. Leveraging the adsorption models of different PCEs on CaMMT and utilizing the hydrogen (H) atom as an example, the Mulliken population analysis for various PCEs is presented in Table 1, while an error bar chart for the charge of H atoms after adsorption is shown in Figure 8.

As presented in Figure 8, the variance between different PCEs is not significant at H (31), H (43) and H (48). There is a statistically significant and relatively large difference in magnitudes for different PCEs at H (14) and H (41) and also with the changes in Mulliken charge populations. APEG had a higher number of electron transfers at H (5), H (21), H (41) and H (57) [37,38], and the total number of transferred electrons (absolute value) was 0.648 in APEG. The number of transferred electrons in APEG was more pronounced compared to IPEG and HPEG, aligning with the DOS analysis conducted earlier. The heightened changes in the DOS corresponded to more substantial alterations in transferred electrons, indicating a stronger adsorption between the PCEs and CaMMT.

## 3. Models and the Calculation Method

Density functional theory (DFT) has been widely used in material synthesis and surface adsorption studies. In this study, all DFT calculations were performed using the DMol^3^ package incorporated in the Material Studio 8.0 (MS) software, which can be used to study adsorption structures, reaction energies, bond strengths, molecular orbitals, etc. The equilibrium configuration at the lowest energy was determined by geometry optimization using the Becke–Perdew (BP) correction approximation under the generalized gradient approximation (GGA); the smearing value was set to 0.005 unless otherwise specified [39,40,41]. The density of state, electronic structures and charge distribution are presented in the following calculations.

Based on previous studies, we concluded that the admixtures exhibit a higher affinity for adsorption onto montmorillonite compared to other minerals, so we adopt CaMMT as the subject of investigation, with calculations centered on the CaMMT (020) surface [12,24]. The CH was adopted using a (2 × 2 × 2) surface crystal model, and the CH (101) surface was used for the following discussion [24].

**Figure 9:**
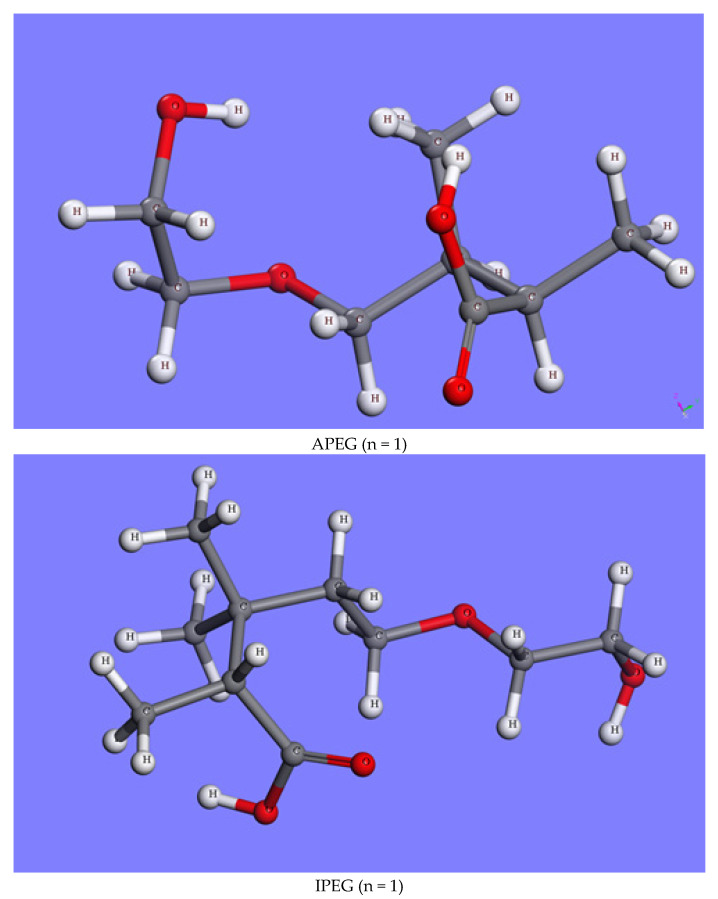

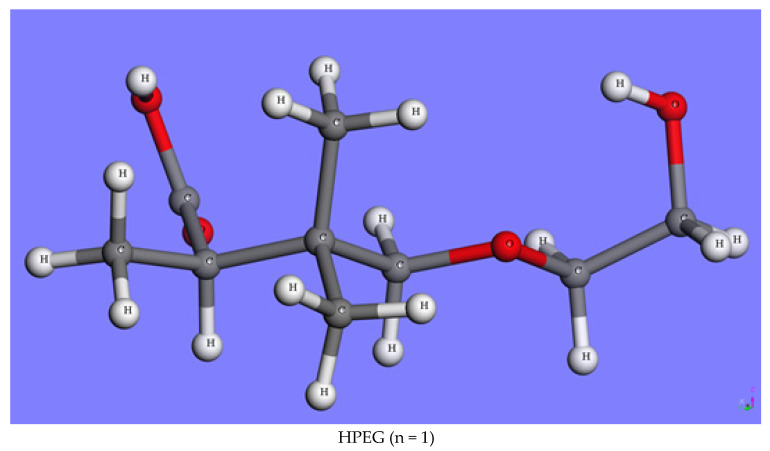
Models of different PCEs after geometry optimization.

Several PCEs with different side-chain lengths were explored, as shown in Figure 1. Taking a polymerization degree of n = 1 as an example, the PCE models after geometry optimization are shown in Figure 9 [37,42].

The adsorption energies were calculated using the following equations:

*E_ads_* = *E_total_*_*(PCEs on*_
*_CaMMT_*_)_ − (*E_PCEs_* + *E_CaMMT_*) (1)

*E_ads_* = *E_total_*_(*PCEs on Ca*(*OH*)2)_ − (*E_PCEs_* +*E_Ca_*_(*OH*)2_) (2)

where *E_ads_* is the adsorption energy; *E_total_* _(*PCEs on*_ *_CaMMT_*_)_ and *E_tota__l_*_(*PCEs on Ca*(*OH*)2)_ are the total energies after adsorption on CaMMT and the hydration products CH. *E_PCEs_*, *E_CaMMT_*, *E_Ca_*_(*OH*)2_ are the respective energies before adsorption. A lower adsorption energy indicates a higher likelihood of adsorption [2,31,36,43].

## 4. Conclusions

This study investigated the preferential adsorption and compatibility of different PCEs with clay and hydration products from the perspective of adsorption energies. Combined with the electron gain and loss analyses, the interaction mechanism of PCEs with clay and hydration products was elucidated. The primary conclusions are as follows:

(1) Competitive adsorption between CaMMT and CH was evident when comparing the adsorption energies of PCEs with different degrees of polymerization. Given that the adsorption energy on CaMMT is lower than that on CH, the PCEs exhibited a preference for adsorption on clay.
(2) For the PCEs studied in this article, the adsorption difficulty of PCEs on CaMMT increased gradually with the degree of polymerization, with a less pronounced effect observed for the adsorption of HPEGs. When n ≥ 10, the adsorption energy remained negative, indicating that the adsorption on CaMMT was more likely to occur.
(3) After adsorption with PCEs, the DOS of CaMMT may undergo varying degrees of change. The peak values of APEG and IPEG are 46.65 and 46.55 electrons/eV, respectively, higher than that before the adsorption, while the peak area is also larger for APEG at n = 1. The total number of transferred electrons in APEG was 0.648, significantly exceeding those in IPEG and HPEG. The stronger changes in the density of states corresponded to more pronounced alterations in the transferred electrons, signifying a more substantial adsorption between the PCEs and CaMMT. Ultimately, these findings offer a crucial theoretical foundation for the anti-clay modification and structural design of mud-resistant PCEs.

## Figures and Tables

**Table 1:** Comparison of the Mulliken charge populations for different PCEs.

Before and after Adsorption				
Atoms	CaMMT	CaMMT + APEG (n = 1)	CaMMT + IPEG (n = 1)	CaMMT + HPEG (n = 1)
H (5)	0.479	0.415	0.454	0.475
H (14)	0.442	0.397	0.516	0.493
H (15)	0.003	−0.026	0.019	−0.014
H (16)	0.496	0.445	0.471	0.491
H (21)	0.479	0.407	0.469	0.446
H (30)	0.442	0.458	0.367	0.380
H (31)	0.003	0.011	−0.005	0.003
H (32)	0.496	0.441	0.457	0.452
H (41)	0.404	0.515	0.447	0.451
H (42)	−0.000	−0.034	0.013	−0.008
H (43)	0.421	0.434	0.406	0.428
H (48)	−0.003	−0.010	0.005	−0.005
H (57)	0.404	0.459	0.416	0.424
H (58)	−0.000	0.028	−0.013	0.007
H (59)	0.421	0.456	0.415	0.410
H (64)	−0.003	0.022	−0.018	0.014

## Data Availability

The data presented in this study are available on request from the corresponding author. The data are not publicly available due to our following related articles are currently in the submission process.

## Abbreviations

PCE	Polycarboxylic superplasticizers
HPEG	methylallyl polyoxyethylene ether
DOS	density of states
APEG	allyl polyethylene ether
IPEG	isopentenol polyoxyethylene ether
DFT	Density functional theory
MS	Material Studio
BP	Becke–Perdew
GGA	generalized gradient approximation
TDOS	total density of states
